# MicroRNAs: Emerging Regulators of Metastatic Bone Disease in Breast Cancer

**DOI:** 10.3390/cancers14030729

**Published:** 2022-01-30

**Authors:** Marie-Therese Haider, Daniel J. Smit, Hanna Taipaleenmäki

**Affiliations:** 1 Molecular Skeletal Biology Laboratory, Department of Trauma and Orthopedic Surgery, University Medical Center Hamburg-Eppendorf, 20246 Hamburg, Germany; m.haider@uke.de; 2 Institute of Biochemistry and Signal Transduction, University Medical Center Hamburg-Eppendorf, 20246 Hamburg, Germany; d.smit@uke.de; 3 Institute of Musculoskeletal Medicine, University Hospital, LMU Munich, 82152 Planegg-Martinsried, Germany; 4 Musculoskeletal University Center Munich, University Hospital, LMU Munich, 82152 Planegg-Martinsried, Germany

**Keywords:** microRNA, bone metastasis, breast cancer, bone microenvironment, targeted therapy

## Abstract

**Simple Summary:**

MicroRNAs (miRNAs) are short RNA molecules that can interfere with messenger RNA and thus influence protein translation. In recent years, it has been revealed that miRNAs are also involved in carcinogenesis. However, the effect of each miRNA can differ significantly, and they may exhibit pro-tumorigenic or anti-tumorigenic properties. Breast cancer is one of the most common cancer entity in women and distant metastases are frequently observed in the skeleton. The progression of breast cancer bone metastasis largely depends on the interaction of tumor cells and cells of the bone microenvironment. In this review, we summarize the current findings related to miRNAs in metastatic bone disease with a focus on breast cancer. This review emphasizes the impact of miRNAs on both cancer cells and key cells of the bone microenvironment. Additionally, we discuss the potential use of miRNAs as a therapeutic target and elaborate advantages and hurdles of miRNA treatment.

**Abstract:**

Bone metastasis is a frequent complication in patients with advanced breast cancer. Once in the bone, cancer cells disrupt the tightly regulated cellular balance within the bone microenvironment, leading to excessive bone destruction and further tumor growth. Physiological and pathological interactions in the bone marrow are mediated by cell–cell contacts and secreted molecules that include soluble proteins as well as RNA molecules. MicroRNAs (miRNAs) are short non-coding RNAs that post-transcriptionally interfere with their target messenger RNA (mRNA) and subsequently reduce protein abundance. Since their discovery, miRNAs have been identified as critical regulators of physiological and pathological processes, including breast cancer and associated metastatic bone disease. Depending on their targets, miRNAs can exhibit pro-tumorigenic or anti-tumorigenic functions and serve as diagnostic and prognostic biomarkers. These properties have encouraged pre-clinical and clinical development programs to investigate miRNAs as biomarkers and therapeutic targets in various diseases, including metastatic cancers. In this review, we discuss the role of miRNAs in metastatic bone disease with a focus on breast cancer and the bone microenvironment and elaborate on their potential use for diagnostic and therapeutic purposes in metastatic bone disease and beyond.

## 1. Introduction

Breast cancer is the most commonly diagnosed cancer in women worldwide [1]. Increased awareness, improved screening methods, and novel treatment strategies have had a significant impact on disease management, and survival rates for patients with primary breast cancer are now above 90% [2]. Nevertheless, breast cancer remains the leading cause of cancer-related deaths in female patients [1], with the majority of cancer deaths being a consequence of metastatic disease [3]. About 70% of patients with advanced breast cancer will develop metastases in the skeleton, making bone the most frequent site of breast cancer metastasis [4]. Patients suffering from breast cancer bone metastasis are confronted with a tremendous reduction in quality of life, predominantly due to the accelerated cancer-induced bone loss. They often suffer from skeletal-related events (SREs) such as bone pain, spinal cord compression, fractures, and consequently increased morbidity [5].

The establishment of metastatic disease requires several sequential steps, including the detachment of cancer cells from the primary tumor, intravasation and circulation in the blood stream, followed by extravasation at secondary organs, adaptation to the new environment, and, ultimately, proliferation and the formation of overt metastasis [6]. Disseminated breast cancer cells that home to bone arrive in a heterogenous microenvironment that comprises several cell types originating from either hematopoietic or mesenchymal stem cells (HSCs or MSCs, respectively). Briefly, bone-resorbing osteoclasts, which are derived from HSCs, and bone-forming osteoblasts, derived from MSCs, maintain skeletal integrity through a tightly balanced remodeling cycle [7]. Key signaling pathways that regulate osteoclast and osteoblast activity involve the receptor activator of nuclear factor-κB (RANK)/RANK ligand (RANKL)/osteoprotegerin (OPG) axis and canonical Wnt signaling [8]. Additionally, the bone marrow comprises a dense vascular system that couples osteogenesis and angiogenesis [9]. Tight interactions between bone cells and disseminated tumor cells (DTCs) are critical for successful establishment of bone metastasis. Tumor-supportive environments, so-called “niches”, are thought to regulate tumor cell homing, dormancy, and colonization at secondary sites [10,11,12,13,14]. Over the last years, significant progress has been made in identifying and characterizing the cellular and molecular composition of the bone metastasis niche [14,15,16,17,18], which includes the HSC, endosteal (osteoblasts, osteoclasts, fibroblasts), and vascular (endothelial cells) niches [14,16]. However, several other cell types that are present in the bone, such as adipocytes, megakaryocytes, and immune cells, have been reported to regulate metastatic breast cancer growth in bone [17,19,20]. The precise location of these niches remains to be defined; similarly, the extent to which these niches overlap and/or interact remains challenging to elaborate. 

## 2. The Bone Microenvironment as a Therapeutic Target in Breast Cancer Bone Metastasis

Once disseminated breast cancer cells proliferate in bone, the tumor–bone cell interactions result in accelerated osteoclast-mediated bone resorption, a key characteristic of the disease. Briefly, tumor cell-derived factors (e.g., parathyroid hormone-related protein (PTHrP) and interleukin 11 (IL-11)) alter the RANKL/OPG ratio in favor of osteoclast activity [21,22]. During the increased osteolysis, tumor growth-promoting factors are released from the bone matrix (e.g., bone morphogenetic proteins (BMPs), insulin-like growth factor 1 (IGF-1), and transforming growth factor-beta1 (TGF-β1)), resulting in a feedforward loop referred to as the vicious cycle of bone metastasis [21,22]. Osteoclasts are key drivers of the breast-cancer-induced osteolysis; therefore, standard-of-care treatment includes, besides conventional radiation and chemotherapy, agents that inhibit excessive bone resorption [23]. In clinical practice, bisphosphonates (e.g., Zoledronic acid) and Denosumab, an antibody against RANKL, are approved for treatment, while several other osteoclast-modifying agents are still under investigation (e.g., mTOR-inhibitors, Src-inhibitors, cathepsin K-inhibitors) [23,24,25,26]. However, these treatments are only palliative, and the disease remains incurable. In order to counteract the cancer-induced osteolysis, bone-anabolic agents (e.g., Romosozumab, an antibody against Sclerostin [27]), which are used in clinical practice to treat osteoporosis, are currently emerging as promising treatment approaches [28]. Indeed, sclerostin-antibody treatment altered the number and activity of osteoblasts and osteoclasts in vivo, protected against cancer-induced bone destruction, and reduced the bone-metastatic burden in mouse models of breast cancer bone metastasis [29].

Despite these recent advances, disrupting the tight interaction between DTCs and cells of the bone microenvironment remains a challenge that has yet to be overcome. Besides direct cell–cell contact, pathological crosstalk between tumor cells and bone cells is mediated via secreted factors. In this context, microRNAs, small non-coding RNAs that can be transferred between cell types, e.g., via extracellular vesicles (EVs) and exosomes [30,31,32], have been suggested as potential therapeutic targets to intervene with the vicious cycle of bone metastasis.

## 3. MicroRNAs (miRNAs)

**Figure 1:**
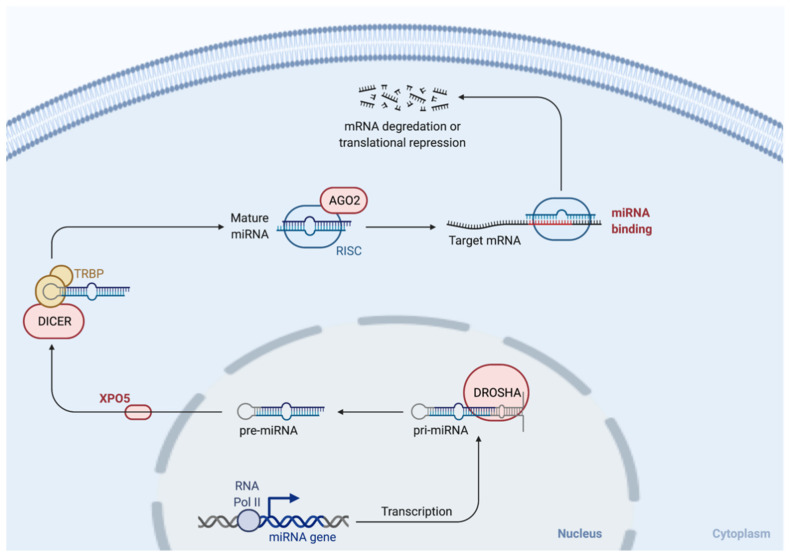
miRNA biogenesis. Following the creation of a pri-miRNA transcript, a complex that contains an RNA-binding protein (DiGeorge Syndrome Critical Region 8 (DGCR8)) and the ribonuclease III enzyme, Drosha, is formed [44]. Pre-miRNAs are formed upon the cleavage of pri-miRNA by Drosha [44]. This process takes place in the nucleus. Following exportation to the cytoplasm by exportin 5 (XPO5), Dicer, another ribonuclease III enzyme, processes the pre-miRNA into its mature form [45]. miRNAs are further processed by the Argonaute (AGO) family of proteins to form an RNA-induced silencing complex (RISC) [45]. Finally, unwinding of the miRNA duplex takes place, enabling the binding of the miRNA to its target mRNA with consequent degradation or transcriptional repression [44,45].

miRNAs are small (~20 nucleotides in length), non-coding RNA molecules that post-transcriptionally control gene expression [33]. Mechanistically, miRNAs bind to their complementary sequences on the 3′ untranslated region (UTR) of their target mRNA and consequently repress the production of the target protein [33,34]. miRNA biogenesis involves several sequential steps, including the generation of primary miRNAs (pri-miRNA) and precursor miRNAs (pre-miRNA, around 60–70 nucleotides), with the subsequent release of mature miRNAs [35]. These processes involve cleaving steps that are performed by Drosha in the nucleus and Dicer in the cytoplasm, two ribonuclease III endonucleases [35,36,37] (Figure 1). Since their original discovery in *C. elegans* in 1993 [33], a great number of miRNAs have been reported and characterized. Functionally, miRNAs are crucial in regulating physiological cell processes, including differentiation, proliferation, migration, and apoptosis [38,39]. miRNAs are dysregulated in several cancers [36], including breast cancer [40]. Studies have now also reported unique miRNA expression patterns depending on the breast cancer subtype [41,42]. Importantly, miRNAs can act as both tumor suppressors and oncogenes, depending on their target gene, and the expression of miRNA clusters with both pro- and anti-tumorigenic roles has been reported in breast cancer [43].

## 4. miRNAs in Breast Cancer Bone Metastasis

**Figure 2:**
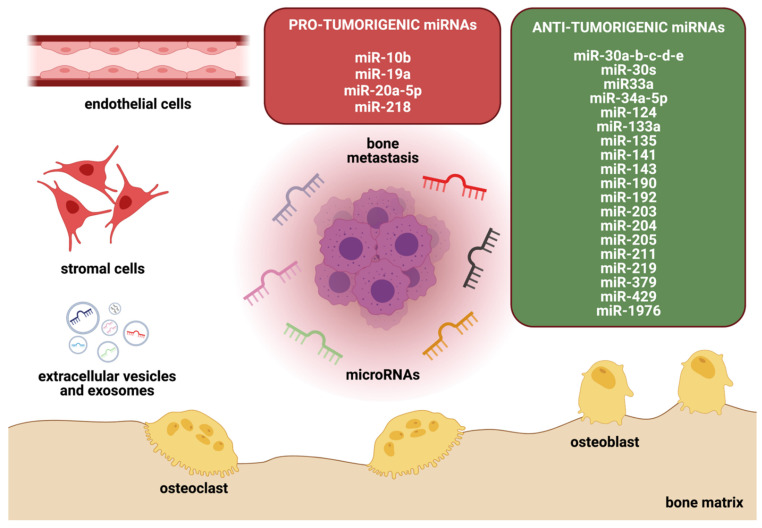
miRNAs in the bone metastatic environment. The bone microenvironment consists of several cell types, including osteoblasts, osteoclasts, stromal cells, and endothelial cells, that orchestrate the tightly regulated bone metabolism. Cancer- and bone-cell-derived miRNAs can either promote or suppress metastatic outgrowth of tumor cells within the bone. The figure depicts the role of miRNAs in breast cancer bone metastasis and groups them according to their influence on tumor growth.

Tightly regulated crosstalk of bone and tumor cells drives the progression of breast cancer growth in bone. miRNAs have been shown to affect both bone and tumor cells [32,46,47,48], highlighting their capability to interfere with the vicious cycle of bone metastasis (Figure 2). Indeed, altered expression of miRNAs has been associated with disease progression and clinical outcome in breast cancer patients, and they are emerging as attractive non-invasive clinical biomarkers [49,50]. Although several established biomarkers for breast cancer diagnosis (e.g., CA 15-3 and CEA) exist, the combination of two or more tumor markers with miRNAs has been shown to increase their diagnostic value [51,52]. A study by Zaleski et al. demonstrated an improvement in both the sensitivity and specificity of breast cancer diagnosis when using miR-34a in addition to the well-established breast cancer tumor marker CA15-3 [52]. Furthermore, lower levels of miR-34a were observed in patients suffering from breast cancer in comparison to benign breast disease and healthy controls. These data underline the potential use of miR-34a in breast cancer diagnosis, as well as differential diagnosis of malignant and benign breast disease. In the same study, Zaleski et al. found a correlation between miR-34a and the Union for International Cancer Control (UICC) stage. The authors observed lower levels of miR-34a in breast cancer patients of UICC stage II or higher, underlining the potential role of miRNAs as prognostic biomarkers in breast cancer [52]. Thus, the significance of miRNAs is not limited to diagnosis; they may also contribute as prognostic biomarkers [53], as miR-10b was one of the first identified miRNAs highly expressed in metastatic breast cancer [54]. High expression levels of miR-10b were observed in breast cancer patients with lymphatic node metastasis [55], as well as in patients with distant metastases in the bone [56] and brain [57]. Furthermore, studies have shown that lower levels of miR-124 in primary breast cancer tissues correlate with shorter bone-metastasis-free survival in breast cancer patients [46]. As another example, miR-218 serum levels have been shown to be elevated in patients with breast cancer bone metastasis when compared to those in patients without metastasis [32]. Similarly, altered miR-124 and miR-218 expression in breast cancer cells has been associated with increased aggressiveness in vitro and in vivo [46,58,59].

### 4.1. Direct Effects of miRNAs on Breast Cancer Cells and Metastasis

Extensive evidence indicates that miRNAs directly affect breast cancer cell behavior and bone metastasis progression. For example, knockdown of miR-1976 in breast cancer cell lines stimulated migration, invasion, and adhesion in vitro when compared to a control [60]. Similarly, lack of miR-1976 promoted epithelial-to-mesenchymal transition (EMT), a key step in metastasis establishment [61], and enhanced cancer stem cell (CSC) properties [60]. Opposing results were observed when cells were transfected with miR-1976 mimics, and the authors attributed a reduced presence of lung metastasis in vivo to miR-1976-induced alterations in EMT and the CSC pool [60]. Similarly, miR-429 has been shown to reduce proliferation, migration, and invasion, as well as EMT, in breast cancer cells in vitro and to inhibit bone metastasis in vivo [62]. A reduced invasion capacity was also observed in the MDA-B02 bone metastatic breast cancer cell line upon overexpression of miR-30b-d, miR-30b-c, or miR-30a-b-c-d-e [63]. Others have shown that transglutaminase 2 (TG2) downregulates miR-205 in breast cancer cells and thereby promotes bone metastasis [64]. Overexpression of miR-143 in breast cancer cells reduced cell viability, migration, and invasion in vitro through targeting mitogen-activated protein kinase 3 [65]. In contrast, others have shown that overexpression of miR-20a-5p stimulates the migration and invasion of breast cancer cells [66]. Studies have also suggested that miR-34a-5p regulates Met expression in breast carcinomas and, thus, progression to metastasis in bone [67]. Met receptor and its ligand hepatocyte growth factor (HGF) are involved in several cellular signaling pathways that regulate proliferation, migration, and invasion, and aberrant Met signaling has been reported in several types of cancer [68]. Indeed, an inverse correlation of miR-34a-5p and the tyrosine kinase receptor Met in breast cancer bone metastasis has been reported [67]. When associated with Met/HGF, miR-34a-5p has thus been suggested as a diagnostic marker predicting poor prognosis [67]. Furthermore, overexpression of miR-203 and miR-135 reduced the migration, proliferation, and viability of breast cancer cells in vitro, with reduced tumor growth in bone observed in vivo [69]. 

#### Osteomimicry-Related Genes in Breast Cancer Cells Are Altered by miRNAs

In order to increase their chance of survival in bone, disseminated breast cancer cells are capable of acquiring a bone-cell-like phenotype, a process known as osteomimicry [70]. Osteomimicry factors expressed by breast cancer cells that home to bone include, for example, Runt-related transcription factor 2 (Runx2), Bone Morphogenetic Proteins (BMPs), Alkaline Phosphatase (ALP), PTHrP, RANKL, or OPG—reviewed in great detail in [71]. Interestingly, miRNAs can affect the expression of several osteomimicry-related genes in breast cancer cells, which could account for their metastasis regulatory function [58,63]. For example, overexpression of miR-30s in MDA-B02 cells reduced osteomimetic genes, including CX43 and CDH11, as well as Dickkopf-related protein 1 (DKK1) [63]. On the other hand, miR-218 increased the expression of bone sialoprotein (BSP), osteopontin (OPN), and a chemokine receptor, CXCR4, in MDA-MB-231 breast cancer cells [58], suggesting that miR-218 supports osteomimicry and, thus, breast cancer cells homing to bone. Furthermore, miR-218 expression in breast cancer cells was associated with elevated Wnt-signaling. Compared to MCF10A breast epithelial cells, metastatic MDA-MB-231 breast cancer cells expressed higher levels of miR-218 and Wnt target genes LEF1 and TCF-4 [58]. In osteoblasts, miR-218 induced and stimulated differentiation, and it induced Wnt signaling by targeting DKK2, Sost, and Sfrp2 [58]. These studies suggest a miR-218/Wnt signaling loop between breast cancer cells and osteoblasts that supports breast cancer bone metastasis [58]. Indeed, overexpression of miR-218 in breast cancer cells promoted osteolytic disease in vivo, while antagonizing miR-218 attenuated tumor growth and bone destruction [59].

Runx2 has been shown to be a direct target of several miRNAs. miR-30 family members reduced the expression of Runx2 in MDA-MB-231 breast cancer cells [63]. Others have shown that ectopic expression of miR-135 and miR-203 in combination with systemic administration of miR-135 and miR-203 reduces orthotopic tumor growth and spontaneous metastasis of MDA-MB231 cells to bone in vivo [69]. Consistently, reduced tumor growth in bone was observed when cells expressing miR-135 and miR-203 were directly injected into the tibiae [69]. This was accompanied by reduced cancer-induced osteolysis and fewer TRAP^+^ osteoclasts. The authors suggest reduced expression of Runx2 and related target genes, including IL-11, MMP13, and PThrP, in breast cancer cells in the presence of miR-135 and miR-203 as a working mechanism [69].

### 4.2. miRNAs Disrupting the Tumor Cell–Bone Cell Crosstalk

**Figure 3:**
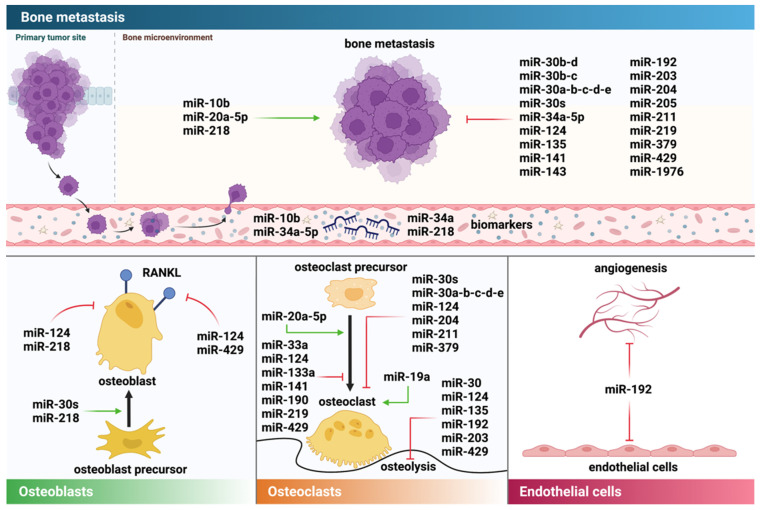
Direct and indirect roles of miRNAs in bone metastasis. miRNAs can affect cancer cells directly, either promoting (green arrows) or inhibiting (red arrows) tumor growth as depicted in the top panel. In addition, miRNAs affect other cells in the bone microenvironment, including osteoblasts, osteoclasts, and endothelial cells.

The studies described in the previous section report direct effects of miRNAs on tumor cell behavior (e.g., migration, invasion, EMT). However, miRNAs can also indirectly affect tumor growth via effects on cells of the tumor/bone microenvironment (Figure 3). The bone microenvironment consists of various cell types, including bone-specific cells such as osteoclasts, osteoblasts, stromal cells, and endothelial cells [16]. A complex signaling network mediates the crosstalk of the plethora of cells within the bone. Especially over the past years, the bone microenvironment has gained tremendous attention, as research suggests an extensive interplay between tumor cells and cells of the tumor microenvironment [14,16]. In the following sections we review the effects of miRNAs on key players within the bone microenvironment. 

#### 4.2.1. Osteoclasts

Given the osteolytic nature of breast cancer bone metastases, the role of osteoclasts in metastatic bone disease has been extensively studied. Various miRNAs have been discovered as important players in this process. For instance, lentivirus-mediated restoration of miR-124 in breast cancer cells reduced metastatic burden and osteolysis in hind limbs of mice when compared to a control [46]. Similar results were observed when mice received treatment with ago-miR-124 after tumor cell injection [46]. Reduced osteoclast number and activity are suggested to be at least partially responsible for the reduced metastatic burden in vivo. Indeed, complementary mechanistic in vitro studies support this hypothesis, as conditioned medium from miR-124-transfected breast cancer cells reduced the viability and differentiation of osteoclasts in vitro [46]. Guo and colleagues showed that breast-cancer-cell-derived exosomes containing miR-20a-5p stimulated the proliferation of bone marrow macrophages and differentiation into osteoclasts in vitro [66]. Recently, Wu et al. reported that exosomal miR-19a not only is a factor secreted by ER+ bone metastatic breast cancer cells, but also mediates osteolysis by creating an osteoclast-enriched environment within the bone in the presence of integrin-binding sialoprotein [72]. 

miRNAs can also directly affect osteoclast differentiation. For instance, ectopic expression of miR-141, miR-190, miR-219, miR-33a, and miR-133a reduced osteoclast differentiation and/or activity in vitro [73]. Combined ectopic expression of miR-141/190/219 was even more effective in reducing osteoclast differentiation and activity than single agents; these effects were further enhanced upon the addition of zoledronic acid, a standard-of-care agent used to ease cancer-induced bone disease [73]. Similarly, systemic miRNA treatment resulted in increased trabecular bone volume in BALB/c mice in vivo [73]. Additionally, miR-141 and miR-219 reduced bone metastasis of SCP28 cells in vivo [73].

Increased levels of matrix metalloproteinase (MMP)-13 have been shown to stimulate osteoclast activity in the metastatic bone environment [74], suggesting that MMPs mediate tumor cell–bone cell communication in the metastatic environment. Conditioned medium from breast cancer cells transfected with miR-124 inhibited the expression of MMP-13 in MC3T3 osteoblasts when compared to a control [46], which could, further on, indirectly reduce osteoclast activity and have consequences on the establishment of breast cancer bone metastasis. In agreement, miR-203 and miR-135 reduced the expression of MMP-13 in breast cancer cells [69], which could partially account for the reduced bone metastases in mouse models in these studies. Others have shown that breast-cancer-cell-derived miR-429 reduces osteoclast differentiation in vitro via MMP-9 and V-crk sarcoma virus CT10 oncogene homolog-like (CrkL) [48]. In vivo, mice injected with miR-429-transfected breast cancer cells had reduced cancer-induced osteolysis when compared to a control, and histological analysis of the bone metastases showed a reduction in both CrKL and MMP-9 expression in the miR-429 group [48]. Others have shown that overexpression of miR-20a-5p in MDA-MB-231 breast cancer cells increases the expression of MMP-2 and MMP-9, which is associated with increased migration and invasion in vitro [66]. In addition, breast-cancer-cell-derived miR-20a-5p was able to stimulate osteoclastogenesis in vitro [66]. 

Interleukins (ILs) have been identified as key regulatory soluble factors that mediate the tumor–bone cell interaction in breast cancer bone metastasis [75]. Several studies have shown that ILs affect the function and maturation of both osteoblasts and osteoclasts [76,77,78]. The majority of the published literature on the matter reports osteoclast stimulatory effects of ILs [79,80,81,82], which highlights them as a therapeutic target in breast-cancer-mediated osteolysis. Studies by Cai and colleagues have attributed the anti-metastatic effects of miR-124 to downregulation of IL-11 and identified IL-11 as a direct downstream target of miR-124 [46]. Briefly, in these studies, mice injected with control cells had significantly increased metastatic burden and osteolysis in hind limbs when compared to mice injected with MDA-MB-231-miR-124 cells, with similar results observed when using ago-miR-124 treatment [46]. The reduced metastatic burden was accompanied by reduced osteoclast number and activity in vivo. Complementary in vitro studies, in which conditioned medium from miR-124-transfected breast cancer cells reduced the viability and differentiation of osteoclasts, support this hypothesis [46]. Importantly, breast cancer cells expressing miR-124 had significantly lower mRNA and protein levels of IL-11 when compared to control [46]. This observation, in addition to complementary in vitro and in vivo studies using IL-11 neutralizing antibodies and recombinant human IL-11, demonstrated that miR-124 induced downregulation of IL-11 is partially responsible for reduced breast cancer bone metastasis [46].

TGF-ß is a key regulator of the vicious cycle of bone metastasis; it is released from the bone matrix during osteolysis and consequently stimulates tumor progression [83]. Studies have shown that TGF-ß increases the secretion of osteoclast-stimulating ILs (IL-11 and IL-8) in breast cancer cells [84]. In this context, miR-204, miR-211, and miR-379 have been identified as key regulators of the TGF-ß-induced production of IL-11 in bone metastatic MDA-MB-231 breast cancer cells [85]. By binding to the IL-11 3′ UTR, these miRNAs reduce IL-11 mRNA and protein secretion [85]. 

Additionally, conditioned medium from bone metastatic MDA-B02-cells that overexpressed miR-30 family members reduced osteoclast formation and differentiation in vitro, potentially through reduced expression of the osteoclast-promoting cytokines IL-11 and IL-8 [63]. Furthermore, conditioned medium from MDA-B02 breast cancer cells stably transfected with miR-30s decreased the formation of TRAP-positive multinucleated osteoclasts in vitro [63]. In vivo, mice injected with MDA-B02-pmiR30a-b-c-d-e tumor cells had reduced osteolytic bone disease, reduced bioluminescence signal, and reduced TRAP+ osteoclasts when compared to a control [63].

#### 4.2.2. Osteoblasts

Studies by Liu et al. reported that overexpression of miR-218 in breast cancer cells supports bone metastasis via direct and indirect effects on osteoblasts [32]. The authors reported two independent mechanisms by which miR-218 could affect breast cancer bone metastasis. First, bone metastatic breast cancer cells secrete EVs that contain elevated levels of miR-218 when compared to the parental cell line [32]. The addition of EVs from MDA-231-miR-218 cells to osteoblast cultures reduced type I collagen mRNA expression and the bone formation marker P1NP—a measure of osteoblast activity—in the medium. Similar results, namely, reduced P1NP serum levels, were observed in vivo upon injection of EVs from MDA-231-miR-218 cells when compared to a control [32]. Interestingly though, no effect on osteoblast differentiation was observed in these experiments [32]. As a second mechanism, the authors suggested that overexpression of miR-218 in breast cancer cells alters the expression and secretion of inhibin beta subunits, which consequently affects procollagen processing in osteoblasts [32]. 

Others have shown that, via reducing DKK1—a key inhibitor of osteoblast differentiation [86]—in breast cancer cells, miR-30s stimulates osteoblast differentiation as compared to a control [63]. Additional key regulatory pathways in bone remodeling as well as cancer-induced bone disease include the RANK/RANKL/OPG axis [87] and Wnt signaling [88]. Several miRNAs have been shown to alter the RANKL/OPG ratio with potential consequences on the progression of breast cancer bone metastasis [46,48]. Both miR-124 and miR-429, in independent studies, decreased RANKL and increased OPG in osteoblasts, leading to an altered RANKL/OPG ratio [46,48].

#### 4.2.3. Further Components of the (Bone) Tumor Microenvironment

Besides the heterogenous cell populations and soluble factors, the tumor microenvironment also comprises the extracellular matrix (ECM). MMPs are proteolytic enzymes that are involved in remodeling and/or degrading the ECM, a requirement for metastasis establishment. Additionally, MMPs mediate several steps of metastasis, including tumor angiogenesis and tumor cell proliferation, migration, and invasion [89,90]. Indeed, studies have shown that miRNAs can affect metastatic breast cancer growth in bone via altering the availability of MMPs [46].

The bone microenvironment is highly vascularized [9], and evidence supports the detrimental role of the bone marrow vascular niche in the initiation and progression of breast cancer bone metastasis [11,14,16,91]. miRNAs are also proposed to be involved in regulating tumor angiogenesis. In mouse models of lung cancer bone metastasis, miR-192 has demonstrated anti-metastatic potential. Mice injected with cancer cells overexpressing miR-192 showed reduced osteolytic bone lesions and decreased metastatic burden in the bones [31]. Interestingly, tumors in the miR-192 group were less vascularized, and in vitro studies showed that miR-192 reduces the migration of HUVEC cells, suggesting that miR-192 exerts anti-metastatic effects via vascular endothelial cells [31]. In these experiments, miRNAs were transferred between cell types via exosome-like vesicles [31]. 

## 5. Therapeutic Implications of miRNAs in Metastatic Bone Disease

As stated before and summarized in Table 1, miRNAs may serve as a potential novel therapeutic targets in cancer as they modify both bone and tumor cells. However, to date, no clinical trials targeting breast cancer or metastatic bone disease have been registered in the clinical trials database (www.clinicaltrials.gov, accessed on 22 November 2021). Nevertheless, a handful of Phase I trials and one Phase II clinical trial have been registered in the clinical trials database, targeting miRNAs or using miRNA mimics as a drug in cancer treatment of leukemia, lymphoma, and other solid cancer entities [92].

Regarding breast cancer and metastatic bone disease, several in vivo studies demonstrated miRNA-mediated effects on the bone microenvironment leading to reduced tumor growth and attenuated osteolytic disease. For example, high levels of miR-30 [63], miR-124 [46], miR-192 [31], and miR-429 [62] have been shown to have a beneficial effect with reduced osteolysis in vivo. In addition to osteolytic lesions, the frequency of bone metastasis or tumor burden in general can be altered by differential miRNA expression. For instance, reduced tumor burden, especially in the bone, mediated by miR-135 and miR-203 has been observed in mice [69]. The identification of disease-specific miRNAs brings us a step forward towards more personalized medicine, utilizing an endogenous molecule.

With respect to breast cancer, Ell and colleagues demonstrated that after systemic application of pre-miR-141 and pre-miR-219, the number of osteoclasts was significantly decreased. Although the analysis revealed that osteoblast differentiation was not affected, the authors could not rule out the possibility that other cells are affected by systemic pre-miRNA treatment [73].

## 6. Future Perspectives

In general, RNA-based medicine has received tremendous attention within the last decade [95]. Beyond mRNA-based drugs, non-coding RNAs, including miRNAs, are amenable for therapeutic development. Although miRNA-based drugs have not reached clinical approval yet, several compounds are in pre-clinical and clinical development. These drugs target various diseases, including cancer, where great progress has been made in recent years. In particular, the fact that miRNAs and other oligonucleotide drugs are adaptable molecules holds great promise in personalized medicine with individual and agile drug design.

However, given their biological properties, there are still potential pitfalls related to miRNA-related medicine. As mentioned before, miRNAs are usually short non-coding RNA molecules with an approximate length of 20 nucleotides. Their small size may be an advantage for drug delivery but may lack target specificity. One potential solution to overcome the low specificity could be a mixture of different miRNAs with the same target to ensure reliable target inhibition. Nonetheless, this approach might also increase the rate of off-target effects and, therefore, potentially also the risk of adverse events. In addition to off-target effects, unwanted and unpredictable side-effects within the complex signaling networks should be considered and extensively investigated. 

Once the obstacles—including off-target effects, tissue specificity, and delivery systems—have been overcome, it can be assumed that treatments using miRNAs as targets will further progress, and novel drug candidates will be developed for diseases with high unmet medical needs, such as metastatic bone disease. Given that miRNAs were first discovered only a few decades ago, miRNA-based drug development is still in its infancy, but the conditions are promising for the development of next-generation miRNA-based drugs. More fundamental and translational studies are needed to better understand the mechanisms of action, as well as potential adverse effects related to miRNAs in various disease conditions.

## 7. Conclusions

The currently available results of preclinical studies suggest an important role of miRNAs in metastatic bone disease. Several miRNAs have already been characterized, and the data indicate a potential novel druggable target in cancer therapy that should be further evaluated in pre-clinical development and clinical trials. 

## Figures and Tables

**Table 1:** miRNAs involved in breast cancer bone metastasis.

MicroRNA	Target	Effect on Bone Metastasis	Reference
miR-10b		Promoting	[56,93,94]
miR-1976	Phosphatidylinositol-4,5-bisphosphate 3-kinase catalytic subunit gamma (PIK3CG)	Inhibiting	[60]
miR-429	V-crk sarcoma virus CT10 oncogene homolog-like (CrkL) and Matrix metalloprotease 9 (MMP-9)	Inhibiting	[62] [32,46,47,48]
miR-30 family	Osteomimicry genes e.g., Cadherin 11 (CDH11) and Integrin Alpha 5 (ITGA5), Interleukins	Inhibiting	[63]
miR-205		Inhibiting	[64]
miR-143	Mitogen-activated protein kinase 3 (MAPK3)	Inhibiting	[65]
miR-20a-5p	SRC Kinase Signaling Inhibitor 1 (SRCIN1)	Promoting	[66]
miR-34a-5p	Met	Inhibiting	[67]
miR-135	Runt-related transcription factor 2 (Runx2)	Inhibiting	[69]
miR-203	Runx2	Inhibiting	[69,93]
miR-124	Interleukin-11 (IL-11)	Inhibiting	[46]
miR-19a	Phosphatase and Tensin homolog (PTEN)	Promoting	[72]
miR-141	Microphthalmia-associated transcription factor (Mitf)	Inhibiting	[73]
miR-219	Mitf, TNF receptor associated factor (Traf-6)	Inhibiting	[73]
miR-204, miR-211, and miR-379	IL-11	Inhibiting	[85]
miR-218	Dickkopf-related protein 2 (DKK2), Secreted frizzled-related protein 2 (sFRP2), Sost	Promoting	[32,58]
miR-192	IL-8, Intercellular Adhesion Molecule (ICAM) and C-X-C Motif Chemokine Ligand 1 (CXCL1)	Inhibiting	[31]
